# Abnormal movements associated with oropharyngeal dysfunction in a child with Chiari I malformation

**DOI:** 10.1186/s12887-014-0294-3

**Published:** 2014-12-10

**Authors:** Stéphanie Berthet, Louis Crevier, Colette Deslandres

**Affiliations:** Department of Pediatric Gastroenterology Hepatology and Nutrition, CHU Sainte Justine, University of Montreal, Montreal, QC Canada; Department of Pediatric Neurosurgery, CHU Sainte Justine, University of Montreal, Montreal, QC Canada; CHU Sainte Justine, 3175, Côte Sainte Catherine, H3T1C5 Montréal, QC Canada

**Keywords:** Chiari I malformation, Oropharyngeal dysfunction, Abnormal movements, Gastroesophageal reflux (GER), Gastroesophageal reflux disease (GERD)

## Abstract

**Background:**

Chiari I malformations (CM I) are rare hindbrain herniations. Dysphagia and other oropharyngeal dysfunctions may be associated with CM I, but to our knowledge, no clinical presentation similar to ours has ever been reported. The purpose of this communication is to draw attention to a unique and atypical clinical presentation of a child with CM I.

**Case presentation:**

A 7-year-old boy was evaluated for a two month history of atypical movements which would occur in the evening, and last for an hour after eating. These stereotypical movements with the head and chest bending forward and to the left side, accompanied by a grimace, were associated with sensation of breath locking without cyanosis. Pain and dysphagia were absent. The neurological examination was normal. The possibility of Sandifer syndrome posturing occurring with gastroesopageal reflux disease was considered but neither pain nor back hyperextension were associated with the atypical movements. Neither proton pump inhibitors (PPI) nor prokinetic agents improved his symptoms.

Upper endoscopy and esophageal biopsy did not reveal eosinophilic esophagitis nor reflux esophagitis. Ear, throat and nose (ENT) exam was normal. A severe gastroparesis was demonstrated on milk scan study. Two 24 hour oesophageal pH probe studies pointed out severe gastroesophageal reflux (GER). High resolution manometric evaluation of the oesophagus revealed normal sphincter pressures and relaxations with no dysmotility of the esophageal body. Electroencephalography and polysomnography were normal. A brain magnetic resonance imaging (MRI) was performed and revealed a CM I: cerebellar tonsils extending to 12 mm, with syringomyelia (D4-D5).

For a long period of time, the child’s abnormal movements were considered to be nothing but tics and the CM I a fortuitous finding. Since the child remained symptomatic despite medical treatment, it was decided to proceed with surgery. One year after the onset of his symptoms, he underwent posterior fossa decompression with upper cervical laminectomy and expansion duroplasty. Postoperative MRI confirmed adequate decompression. His atypical posture and dyspnea completely resolved after surgery and he remains asymptomatic two years later.

**Conclusion:**

Children may have atypical presentations of CM I. Thus, CM I diagnosis should be considered in unexplained atypical oropharyngeal dysfunctions.

## Background

Chiari I malformations (CM I) are rare hindbrain herniations that may be present in children or adults. CM I is characterized by an abnormal position of the cerebellar tonsils, which herniate outside the cranial cavity into the upper cervical canal: this is associated with an obliteration of the subarachnoid spaces at the level of the foramen magnum [1,2]. Anomalies associated with CM I include syringomyelia. CM I can be easily identified on magnetic resonance imaging (MRI) of the cranio-vertebral junction [3]. Tonsillar herniation of 5 mm below the foramen magnum is the most common cut off for radiological diagnosis of CM I [4]. More recently, because of the ease of diagnosis and increased clinical awareness, pediatric cases are increasingly reported [5]. Many studies have reported symptoms such as headaches, scoliosis or neurological troubles which were attributed to compression of neural structures. Dysphagia and other oropharyngeal dysfunctions have also been reported but, to our knowledge, no clinical presentation similar to ours has ever been reported.

The purpose of this communication is to draw attention to a unique and atypical clinical presentation of a child with CM I.

## Case presentation

A 7-year-old boy was evaluated for a two month history of atypical movements presenting in the evening, and lasting an hour after eating. These stereotypical movements with the head and chest bending forward and to the left side, accompanied by a grimace were associated with sensation of breath locking without cyanosis. Pain and dysphagia were absent. The neurological examination was normal. The possibility of Sandifer syndrome posturing occurring with gastroesophageal reflux disease (GERD) was considered but neither pain nor back hyperextension were associated with the atypical movements. PPI did not improve his symptoms. Various prokinetic agents (metoclopramide, motilium, cisapride and erythomycin) were also inefficient.

**Figure 1:**
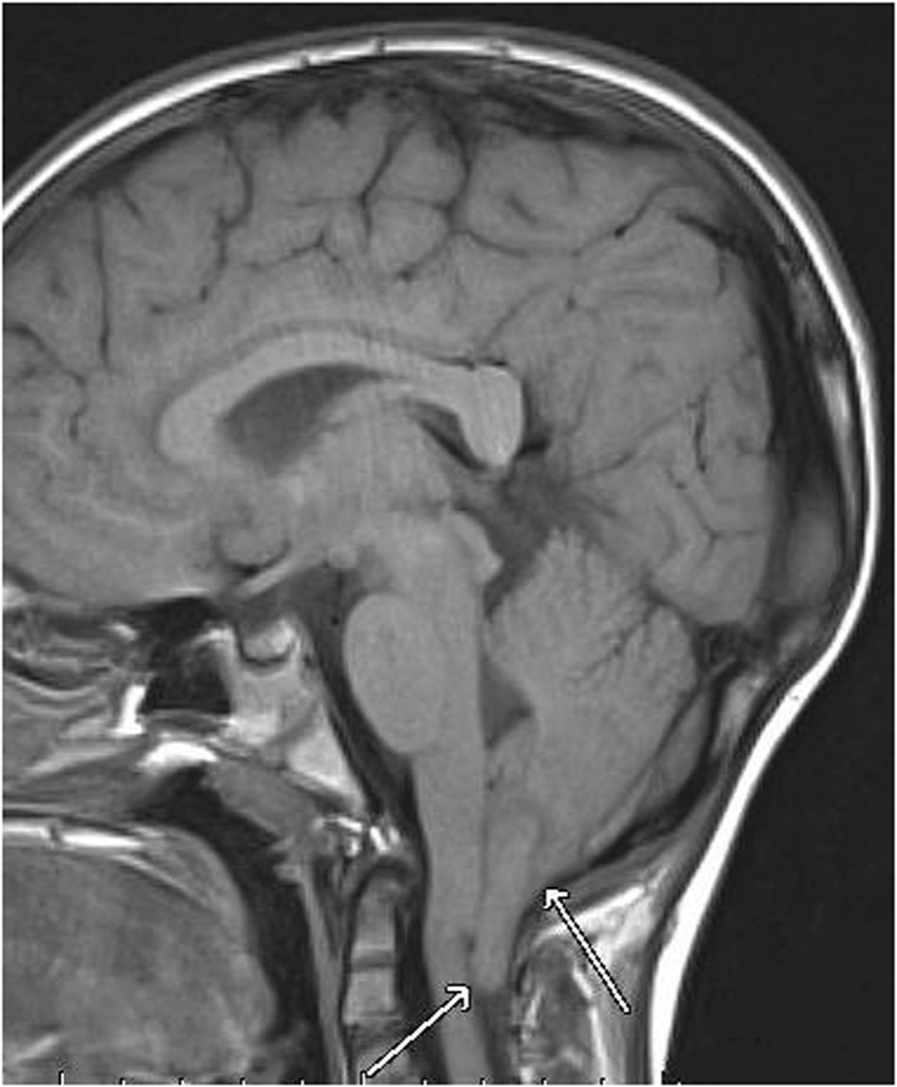
**Cerebellar tonsils herniation on magnetic resonance imaging: Chiari malformation type I.**

Upper endoscopy and esophageal biopsy did not reveal eosinophilic esophagitis or other abnormalities. ENT exam was normal. A severe gastroparesis was demonstrated on milkscan study. Two 24 hour esophageal pH probe studies pointed out severe GER. High resolution manometric evaluation of the oesophagus revealed normal sphincter pressures and relaxations with no dysmotility of the esophageal body. Electroencephalography and polysomnography were normal. Because of the unexplained dyspnea associated with this abnormal posture, a head MRI was performed and revealed a CM I: cerebellar tonsils extending to 12 mm, with syringomyelia (D4-D5) (Figure 1).

For a long period of time, the child abnormal movements were only considered to be tics and the CM I was considered a fortuitous finding. Since the child remained symptomatic despite medical treatment, it was eventually decided to proceed with surgery. The operative procedure was done one year after the onset of his symptoms. He underwent posterior fossa decompression with upper cervical laminectomy and expansion duroplasty. No postoperative complication occurred. Postoperative MRI confirmed adequate decompression. His atypical posture and dyspnea completely resolved in the week after surgery. More than two years after surgery, the child remains asymptomatic. The patient and parents have refused any further invasive testing (such as a control esophageal pH probe study) as the patient was symptom free.

## Discussion

Although CM I is increasingly detected in children [3,5], much remains unknown about its natural history. The pathophysiology of CM I and its associated anomalies have been the subject of considerable debate [1]. Chiari I is a multifactorial condition that is thought to result from a congenital small posterior fossa. Neurologic signs and symptoms may be related directly to a tight foramen magnum associated with the cerebellar tonsillar herniation, with compression and/or distorsion of the medulla and lower cranial nerves.

In adults, the most common clinical symptoms are posterior headaches and/or neck pain exacerbated by Valsava maneuvers [4]. The clinical presentation of young children with CM I differs from that of older children and adults. Albert et al. showed that patients aged 0 to 2 years were much more likely to have oropharyngeal dysfunction, whereas those aged 3 to 5 years were more likely to have syringomyelia, frequently associated with scoliosis [6]. CM I pediatric presentations from published series are reported in Table 1. These retrospective series include both operated and non operated CM I patients: 26 to 37% of the patients [4,7] were asymptomatic with a fortuitous discovery, while the remaining had a variety of neurological symptoms including headaches, ataxia, sensory or motor deficits and lower cranial abnormalities. The most common symptoms were headaches and scoliosis [5].

**Table 1:** **Clinical presentations of Chiari malformation type I in children**

	**Tubbs et al.** [5] **Alabama**	**Benglis et al.** [4] **Miami**	**Caldarelli et al.** [2] **Roma**	**Greenlee et al.** [3] **Iowa**	**Albert et al.** [6] **Iowa**	**Aitken et al.** [7] **San Francisco**	**Park et al.** [1] **Boston**
Study period	1989-2010	1999-2008	1993-2005	1987-2001	1984-2007	1997-1998	1988-1996
No. of children	500	124	30	31	39	51	68
Inclusion criteria	-	no surgery	symptomatic	age <6	age <6	age <20	-
Retrospective study	yes	yes	yes	yes	yes	yes	yes
Age at diagnostis	11	7	5,5	3,5	3,5	11	12
Male %	54	-	40	42	39	-	48
Surgery	500	0	30	25	39	8	68
Asymptomatic %	-	35	0	-	-	37	-
Serious manifestations %	-	-	-	-	-	-	-
Loss of consciousness %	1,2	1,6	-	-	-	4	-
Headache %	40	39	57	23	46	55	63
Neck pain %	-	11	-	-	-	12	-
Ataxia %	4	-	20	-	-	8	16
Sleep apnea %	5	-	20	29	-	-	-
Motor deficit %	10	4	70	3	-	20	45
Sensory deficit %	-	12	36	6	-	6	-
Scoliosis %	18	4	7	23	28	2	16
Dysphagia, oropharyngeal dysfunction %	4	-	7	35	74	-	-
Vomiting %	3	16	3	-	-	-	-
Dyspnea %	1,2	-	-	-	-	-	-
Dysarthria %	5	-	7	-	-	4	-
Abnormal movement	-	-	-	19	-	-	-

- = no value.

Oropharyngeal dysfunction is not frequently reported. In fact, in Tubbs’study on 500 cases of pediatric CM I, oropharyngeal dysfunction only represented 4% of the symptoms [5]. In some studies involving over 100 patients no esophageal symptoms were reported [1,4,7]. Moreover, these dysfunctions are often poorly described, and can manifest with cough, stridor, dysphagia, abnormal vocal cord movement, GERD, aspiration, prolonged feeding, vomiting, sleep apnea or failure to thrive [3,6]. Perkin et al. [8] have reported common dysphagia in patients with CM1 malformation by traction of the lower cranial nerves secondary to the herniation by the CM1 malformation. Dysphagia is associated with a global impairment of all phases of swallowing on videofluoroscopy. As they mention dysphagia may be the presenting symptom in some patients.

Cardi et al. [9] described gastroparesis as a cause of Sandifer syndrome. Indeed gastroparesis may enhance GERD and thus subsequently induce a Sandifer syndrome. Our patient had a very unusual presentation and we initially thought that he presented with an atypical case of Sandifer syndrome as he had well documented severe gastroparesis and GERD. We do not exclude that he might have had pre-existing asymptomatic gastroparesis. Deterioration of his CMI might have worsened his gastroparesis. We were unable to obtain invasive diagnostic procedures (as esophageal pH probe study) after the patient’s surgery but we did obtain a milk scan study a year after surgery which showed improved but persistent gastroparesis in an absolutely symptom free patient. Neurological and gastrointestinal symptoms are frequently associated in different neurological conditions.

Greenlee et al. reported abnormal movements in 6 children with CM I but did not describe them [3]. To our knowledge, no study has reported abnormal movements related to eating in association with CM I.

In conclusion, our patient’s presentation is clearly unique and the total resolution of symptoms following posterior fossa decompression surgery confirms the link between the abnormal postures and CM I. Again, we were unable to perform control studies following surgery due to the patient and the parents’ decision to not perform any further invasive testing.

In the literature, complications occur in only 2.4% of the patients undergoing decompressive surgery for CM I [5]. Spontaneous resolution of childhood CM I has been described in several cases [10]. The Benglis study represents the largest series of pediatric patients with CM I followed without surgery. No new neurological deficits were observed during the follow up period in this population [4], adding to the controversy regarding the indication for surgery [7]. Therefore, pediatric patients with CM I who are not clearly symptomatic and do not have a syrinx or scoliosis, should not undergo surgery [4].

## Conclusion

Symptomatic CM I are being increasingly recognized in young children. The availability of MRI has certainly contributed to this phenomenon. As shown in our case, children may present with atypical manifestations, making CM I a complex clinical diagnostic challenge. CM I should be considered in the differential diagnosis of atypical oropharyngeal dysfunction.

## Consent

Written informed consent was obtained from the patient’s parents and assent was obtained from the patient (as he was too young for a written consent) for publication of this case report and any accompanying images. A copy of the written consent is available for review by the Editor of this journal. The study was approved by the local institutional board.

## Abbreviations

CM 1: Chiari I
PPI: Proton pump inhibitors
ENT: Ear, throat and nose
MRI: Magnetic resonance imaging
GERD: Gastroesophageal reflux disease
GER: Gastroesophageal reflux
